# Neural stem cell spacing questions their self-renewal

**DOI:** 10.18632/aging.101519

**Published:** 2018-08-09

**Authors:** Olga Mineyeva, Alexei Koulakov, Grigori Enikolopov

**Affiliations:** 1 Center for Developmental Genetics and Department of Anesthesiology, Stony Brook University, Stony Brook, NY 11794, USA; 2 Moscow Institute of Physics and Technology, Moscow 123182, Russian Federation; 3 P.K. Anokhin Institute of Normal Physiology, Moscow 125315, Russian Federation; 4 Kurchatov Institute National Research Center, Moscow 123182, Russian Federation; 5 Cold Spring Harbor Laboratory, Cold Spring Harbor, NY 11724, USA

**Keywords:** aging, neural stem cells, adult neurogenesis, symmetric divisions, geometry, distribution, randomness, bias

Adult tissues harbor a limited number of stem cells, using them for growth and tissue repair. This pool of stem cells is inevitably depleted with age – unless, that is, it can be replenished through symmetric stem cell divisions, recruitment or transdifferentiation of other cell types, or supply from other tissues. Of these, self-renewal of stem cells via their symmetric divisions is presumed to be predominant and essential means for preventing the pool’s exhaustion. However, for a number of adult tissues, including the brain, symmetric divisions of stem cells have rarely been formally proven or directly observed.

The question of whether symmetric stem cell division occurs in the brain is vital: while neural stem cells of the hippocampus produce new neurons postnatally, the rate of neurogenesis tapers off with age, possibly contributing to age-related decline in hippocampus-dependent memory in animals and humans (even as the actual scale of a decrease in human neural progenitors with age is up for debate [1]). A likely cause of age-related decline in neurogenesis is the continuous, gradual disposal of the stem cell pool, inherently linked to the new neuron generation – a model initially proposed based on lineage tracing in populations [2] and recently confirmed for individual stem cells by direct observations in the live brain through an implanted window [3]. Asymmetric divisions of stem cells with their subsequent differentiation would eventually lead to the exhaustion of the stem cell pool. Symmetric division could, on the other hand, replenish the pool, providing a more “optimistic” model. Clearly, the modes of neural stem cells’ division have starkly different consequences for the pool’s resilience and, by extension, the prospects for brain repair and rejuvenation.

Thus, researchers are intensely scrutinizing neural stem cell maintenance and division. Most often, symmetric divisions are implied based on the close proximity of stem cells that have been recently engaged in divisions: in this view, detection of two or more stem cells in close vicinity after tagging replicating DNA or upon clonal analysis indicates their birth through symmetric division [4]. This interpretation relies on an inherent assumption: that within the analyzed brain region, the spatial distribution of stem cells and their entry into the division cycle is random. But this assumption has never been directly challenged, and the overall distribution of stem cells in the adult hippocampus may carry a preexisting bias (and, indeed, does, following the dorsoventral axis). In addition, the distribution of cells that have entered the division cycle may itself be biased in a fully different manner, further complicating the assumption of randomness. Why might there be such biases? They may be due, among other possibilities, to nonrandom positioning of the neuroepithelial cells or their progeny during embryonic development, the effect of a blood vessel on location or division of neighboring stem cells, selective elimination during development or adulthood, or non-randomness of recombination upon lineage tracing protocol. No matter the reason, the assumptions that stem cells and their dividing subset are distributed randomly, or that the potential biases in their distribution are similar, may be false and may compromise the conclusions drawn from labeling or clonal analysis experiments.

Therefore, we set out to study the impact of the biases in neural stem cell spatial distribution in the hippocampus. As it is not informative to determine such biases directly, by locating all stem cells and all dividing stem cells, we tackled the problem a different way - by comparing the biases in the distribution of these two sets of cells, to see whether or not they were similar. In other words, one can abstract away from the spatial distributions per se and instead focus on the similarity of the potential biases of these distributions.

**Figure 1:**
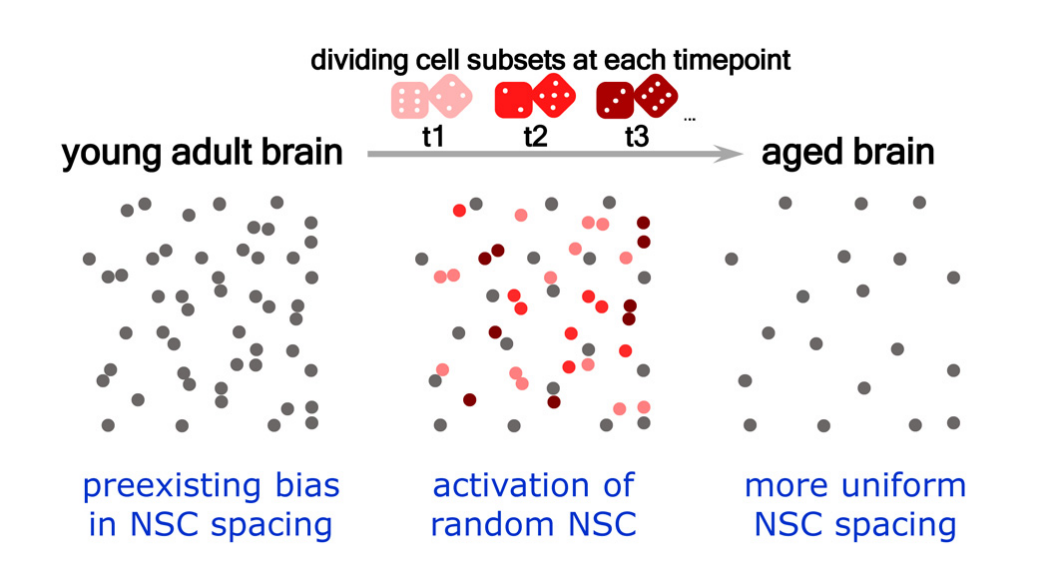
**Distribution of dividing stem cells may reflect pre-existing bias in their spacing, and aging tends to randomize the distribution.** Neural stem cells - grey dots; dividing stem cells at timepoints t1, t2, and t3 – pink, red, and carmine dots. Stem cells deplete with age, a process driven by the activation of their asymmetric divisions, with eventual production of neurons. At each timepoint a subset of stem cells is dividing and some of these are found in adjacent positions which can be perceived as symmetric divisions. However, the rate of division and geometry of cell positioning within each subset is random (symbolized by dice) and the bias of the spacing of the dividing population of stem cells reflects a similar bias in the spacing of all cells. Gradual disappearance of pairs of closely positioned cells evens out the original spacing bias.

Using reporter mice, we generated large data sets on the 3D spatial distribution of the entire pool of radial glia-like neural stem cells in the dentate gyrus of the hippocampus. By tagging dividing cells in the same mice with a nucleotide analog, we also identified the subsets of these cells that have recently undergone divisions, whether symmetric or asymmetric. Then, we asked how the geometries of these two sets compared if either symmetric or asymmetric divisions of neural stem cells are assumed [5]. Remarkably, within the purely asymmetric division model, we found that even when a bias in the distribution of dividing stem cells was observed, it could be explained solely as the pre-existing bias in the distribution of all stem cells, without invoking symmetric division, and hence self-renewal, of neural stem cells (Figure 1).

We then applied the same approach to ask whether the similarity between biases is preserved upon aging, a critical question given the dramatic aged-related stem cell decline. Somewhat unexpectedly, we found that age-related disposal of stem cells tends to even out the spacing between the remaining stem cells in the dentate gyrus, with adjacent cells disappearing at a higher rate; this observation holds true for most subregions and the blades of the dentate gyrus. Underlying mechanisms for this finding are currently unknown but may include juxtapositions of stem cells with the vessel net, with the sources of Shh, Wnt, and Notch signals or GABAergic inputs [6,7], or with other stimuli that would favor closely positioned pairs or clusters of stem cells.

Our findings suggest that asymmetric divisions are the predominant mode of stem cell division in the adult hippocampus. This calls for reevaluating widely held assumptions on the distribution, maintenance, and divisions of neural stem cells, on the possibility of switching modes of division in response to cognitive or emotional stimuli, environment, and disease, and on the prospects of rejuvenation of the ailing nervous system. These findings also emphasize the need for finding new agents and targets for controlling the modes of stem cell division: if neural stem cells are not dividing symmetrically on their own, then we may need to induce that mode of replication to repair the aging or damaged brain.
